# Supplementary material for: Baseline Splenic Volume as a Prognostic Biomarker of FOLFIRI Efficacy and a Surrogate Marker of MDSC Accumulation in Metastatic Colorectal Carcinoma
Source: Cancers (Basel). 2020 May 31;12(6):1429. doi: 10.3390/cancers12061429 (PMC7352427; doi:10.3390/cancers12061429)
Supplement: Supplementary file 1 [file cancers-12-01429-s001.pdf]

cancers MDPI

Supplementary Materials: Baseline Splenic Volume as a Prognostic Biomarker of FOLFIRI Efficacy and a Surrogate Marker of MDSC Accumulation in Metastatic Colorectal Carcinoma

Table S1. Factors associated with overall survival using baseline splenic volume as a binary variable by multivariate Cox analyses.

Variable	HR	95%CI	*p*-Value
Baseline splenic volume >180mL vs ≤180 mL	1.094	[0.819 - 1.461]	0.54
Primary tumor resected yes vs no	0.761	[0.576 - 1.006]	0.06
Number of metastatic sites, >2 vs ≤2	1.743	[1.272 - 2.389]	0.01
Baseline Alcaline Phosphatase,>300 U/L vs ≤300 U/L	2.366	[1.610 - 3.476]	<0.01
Baseline Leukocytes, >10 G/L vs ≤10 G/L	1.500	[1.070 - 2.103]	0.02
Baseline Platelets, >300 G/L vs ≤300 G/L	1.457	[1.092 - 1.945]	0.01

HR, hazard ratio; CI, confidence interval.

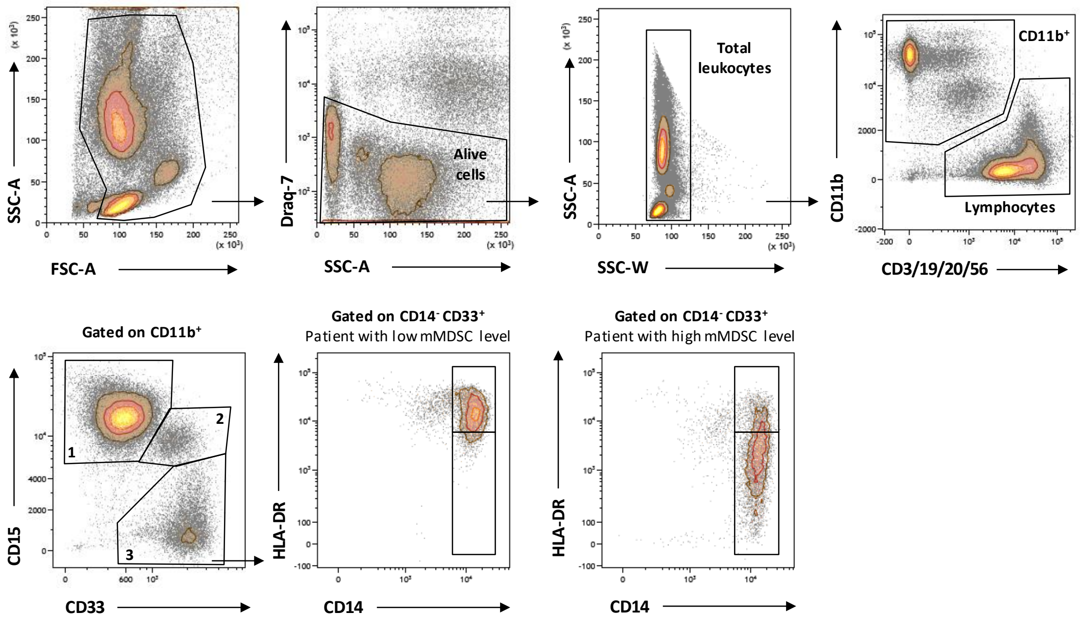

Figure S1. Identification of blood leukocyte subsets by flow cytometry. Singlet live blood CD11b$^+$ Lineage$^-$ (CD3/CD56/CD19/CD20) leukocytes were considered as myeloid cell and CD11b$^-$ Lineage$^+$ as total lymphocytes. Next, we identified (1) granulocytes as CD15$^+$ CD33$^-$, (2) gMDSC as CD15$^+$ CD33$^+$, and (3) monocytic cells as CD15$^-$ CD33$^+$. mMDSC was considered as CD14$^+$ HLA-DR$^{low/neg}$ and mature monocytes as CD14$^+$ HLA-DR^{high}. Representative dot plots of patients with high or low levels of mMDSC are shown.

© 2019 by the authors. Submitted for possible open access publication under the terms and conditions of the Creative Commons Attribution (CC BY) license (http://creativecommons.org/licenses/by/4.0/).
